# Removal of fluoride from coke wastewater by aluminum doped chelating ion-exchange resins: a tertiary treatment

**DOI:** 10.1007/s11356-021-16299-8

**Published:** 2021-09-07

**Authors:** Jesús Rodríguez-Iglesias, Lara Alcalá, Laura Megido, Leonor Castrillón

**Affiliations:** Department of Chemical and Environmental Engineering, Polytechnic School of Engineering, Gijón Campus, University of Oviedo, 33203 Gijón, Spain

**Keywords:** Adsorption kinetics, Chelating resin, Fluoride removal, Freundlich isotherm, Industrial wastewater treatment, Ion exchange, Langmuir isotherm

## Abstract

Coke wastewater is one of the most problematic industrial wastewaters, due to its large volume and complex pollutant load. In this study, ion exchange technology was investigated with the objective of reducing the fluoride content of the effluent from a coke wastewater treatment plant (26.7 mg F^-^/L). Two Al-doped exchange resins with chelating aminomethyl-phosphonic acid and iminodiacetic groups were assessed: Al-doped TP260 and TP207 resins, respectively. The effect of resin dosage, varying from 5 to 25 g/L, was evaluated. F^-^ removal was within the range 57.8–89.3% and 72.0–92.1% for Al-doped TP260 and TP207, respectively. A kinetic study based on a generalized integrated Langmuir kinetic equation fitted the experimental data (R^2^ > 0.98). The parameters of the said kinetics met the optimal conditions for the ion exchange process, which seemed to be more favorable with Al-doped TP260 resin than with Al-doped TP207 resin, using the same resin dosage. Furthermore, the experimental data were well described (R^2^ > 0.98) by Langmuir and Freundlich isotherm models, in agreement with the findings of the kinetic study: the maximum sorption capacity was obtained for the Al-doped TP260 resin.

**Supplementary Information:**

The online version contains supplementary material available at 10.1007/s11356-021-16299-8.

## Introduction

Coke is a product of the distillation of coal and has applications in the iron metallurgy, mineral wool production, non-iron metallurgy, molding, metal sintering, lime kilns, iron blends and carbide production. Worldwide, there are over 560 coke oven plants, more than 70% of them with a production capacity of over 600,000 tonnes per year (Kwiecińska et al. 2017). In 2018, European coking plants used 49 million tonnes of coking coal to produce 37 million tonnes of coke-oven coke (Eurostat 2019).

Coke manufacturing implies several hydro-intensive operations, such as quenching of hot coke, washing the ammonia still, cooling and washing of the coke oven gases, and isolation of the by-products of coke (Ghose 2001). Worldwide, 750 million m^3^ of coke wastewater are generated every year (0.6–1.6 m^3^ of wastewater per every ton of coke) and 92 million m^3^ are generated in Europe (Kwiecińska et al. 2017).

Due to its large volume and complex pollutant load, coke wastewater is one of the most problematic industrial wastewaters, containing organic (organic matter, phenols, polycyclic aromatic hydrocarbons) and inorganic (cyanides, thiocyanates, ammonia, fluorides, sulfides) toxic compounds (Marañón et al. 2008; Pal and Kumar 2014).

Biological treatment is the most widely used alternative on an industrial scale for the removal of organic matter, thiocyanates, phenols, etc. and the transformation of N-NH_4_^+^ into N_2_ (Marañón et al. 2008). Although biological treatments are an established and viable technology for coke wastewater treatment, a tertiary treatment (e.g. chemical treatment and adsorption) is usually required to meet regulations concerning residual pollutant levels and acute colour bodies (Das et al. 2020).

In industrial effluents, high concentrations of F^-^ are usually reduced below 30 mg/L by chemical precipitation, removing fluoride as CaF_2_, MgF_2_ or BaF (Mohapatra et al. 2009). However, after this process, concentrations of fluorides are usually 7–15 mg F^-^/L, which is above acceptable limits (Oke et al. 2011).

In recent years, several treatment technologies have been studied for defluorination of water, such as adsorption (García-Sánchez et al. 2017; Habuda-Stanić et al. 2014), electrocoagulation (Graça et al. 2019; Mena et al. 2019), electrodialysis (Grzegorzek et al. 2020; Wang et al. 2019), ion exchange (Igwegbe et al. 2019; López et al. 2012), membrane distillation (Moran Ayala et al. 2018), nanofiltration (Fatehizadeh et al. 2020; Singh et al. 2016) and reverse osmosis (Damtie et al. 2019; Shen and Schäfer 2014).

High F^-^ removal efficiency, good sorption capacity, simplicity and good flexibility in working conditions are among the main advantages of ion-exchange and adsorption techniques (Dhillon et al. 2016; Zulfiqar et al. 2014). The lack of selectivity, sensitivity and capacity of the conventional ion exchange resins has fostered the development of chelating ion exchangers, which are composed of a polymer matrix covalently bonded to chelating ligands (i.e. ligands with two or more points of attachment for metal atoms) (Sud 2012). Imino-diacetate chelating resins are commonly used to remove certain ions from an aqueous solution and chelating resins containing aminomethyl-phosphonic acid (AMPA) functional groups exhibit high selectivity towards F^-^ (Millar et al. 2017).

Bhatnagar et al. (2011) reviewed the defluorination capacity of more than a hundred materials. Moreover, Table S1 (Online Resource 1) shows the fluoride removal ability of different ion-exchange resins. Oke et al. (2011) found that aluminum-doped chelating resins can efficiently reduce fluoride concentration from 9 mg F^-^/L to less than 1 mg F^-^/L (2.6 g of F^-^ adsorbed per liter of resin). This was in accordance with Pearson’s theory of soft and hard acids and bases, SHAB, that hydrated F^-^ shows strong affinity towards the hard acid polyvalent metal ions (e.g. Al^3+^, Fe^3+^, Ti^4+^…). Based on this, Li et al. (2020) removed fluorides using a chelating resin containing sulfonated monophosphonic acid bifunctional groups (S9570-Fe(III)) and found a combined mechanism of chemical sorption and intraparticle diffusion behind the process kinetics.

Kinetic models (e.g. pseudo-first and pseudo-second order reaction models, the Elovich model and the intraparticle diffusion model with the Weber–Morris equation) and thermodynamic studies based on adsorption isotherm models (e.g. Langmuir, Freundlich, Temkin, Koble-Corrigan and Redlich-Peterson isotherm models) are commonly used in the literature to describe ion exchange processes and thus, to better understand the underlying removal mechanism (Igwegbe et al. 2019; Li et al. 2020).

Notwithstanding its significance, few studies have focused on the disposal of coke oven effluent (Das et al. 2018). This work presents a case study on ion exchange technology to reduce fluoride content (C_0_ = 26.70 mg F^-^/L) in the effluent of a coke wastewater treatment plant (ECWT) to acceptable limits. Based on the literature, the F^-^ removal performance of two commercial cation exchange resins with chelating AMPA and iminodiacetic groups and doped with aluminum was assessed. Experimental data was described by kinetic and isotherm models, using a generalized integrated Langmuir kinetic equation, pseudo-first-order and pseudo-second-order kinetic models and Langmuir and Freundlich isotherm models, respectively. This modelisation allow us to understand the behavior of the resins and describe the defluorination rate, which, in turn, affects the residence time, an important parameter to take into account when scaling the process.

## Materials and methods

### Wastewater

The wastewater used in this work was the effluent of the aerobic biological process with integrated nitrification-denitrification stages used to treat wastewater generated in the coke oven plant (production capacity: 1 million t coke/year) of an integrated steel mill.

Samples of the ECWT were kept in a refrigerator at 4.0 ± 0.1 °C.

### Ion exchange resins

The resins used in the tests were Lewatit TP260 and Lewatit TP207 (Table 1). Lewatit TP260 is a weakly acidic, macroporous cation exchange resin with chelating AMPA groups. It has a high operative capacity and very good mechanical and chemical stability in acid and alkaline environments. Lewatit TP207 resin, sodium form, is a cationic, macroporous, weakly acidic exchange resin with chelating iminodiacetic groups (Lanxess 2020).

**Table 1:** General description and physical and chemical properties of commercial Lewatit TP260 and TP207 (Lanxess 2020)

Parameter	Lewatit TP260	Lewatit TP207
Ionic form as shipped	Na^+^	Na^+^
Functional group	AMPA	Iminodiacetic acid
Matrix	Styrenic	Styrenic
Structure	Macroporous	Macroporous
Total capacity (min) (H^+^ form)	2.4 eq/L	2.0 eq/L
Mean bead size	0.63 ± 0.05 mm	0.61 ± 0.05 mm
Bulk density	720 g/L	700 g/L
Density	1.18 g/mL	1.14 g/mL
Water retention	58–62 wt.%	55–60 wt.%
Stability at pH-range	0–14	0–14

### Experimental methods

#### Preparation of Al-doped exchange resins

The ion exchange resins were functionalised with aluminum before their use to obtain Al^3+^-type chelating resins (see Figure S1 in Online Resource 1).

Firstly, the resin is washed with deionized water and then with a solution of NaF (1 g/L), for at least 1 h of stirring at 290 rpm. Secondly, the resin is washed again with deionized water and doped with Al^3+^ using 5.5%w/v of AlCl_3_ solution (Oke et al. 2011), i.e. 0.41 mol Al^3+/^L, for 1 h at 290 rpm at room temperature. Al^3+^ connects with two points of attachment to the AMPA and iminodiacetic groups of TP260 and TP207, respectively, and with Cl^-^. The Cl^-^ ions are exchanged with F^-^ during the ion exchange process (see Figure S2 in Online Resource 1).

#### Filtration of the effluent of the coke wastewater treatment plant

A filtration process was carried out to remove solids from the ECWT using a 0.45-μm filter before applying the ion exchange process. The pH, total solids (TS), volatile solids (VS), chemical oxygen demand (COD) and five anions (fluorides, chlorides, nitrates, nitrites and sulphates) were determined.

#### Ion exchange batch tests

Ion exchange tests were carried out by mixing 300 mL of ECWT with different amounts of the resin in Erlenmeyer flasks: 1.5, 3.0, 4.5, 5.1, 6.0 and 7.5 g. A SELECTA vibrating shaker (model Vibromatic) was used for this purpose. The stirring was maintained for 15 h at room temperature to ensure that equilibrium was reached (Li et al. 2020). Samples of the treated wastewater were taken for analysis every 5–10 min during the first 120 min to be analysed. After that, samples were extracted at more widely separated intervals. The total amount of wastewater extracted represented less than 10% of the initial volume.

At the end of the test, the solid and liquid phases were separated by sedimentation.

### Analytical methods

The parameters analysed to characterize the wastewater and effluents from the laboratory treatment were pH, TS, VS, COD, fluorides, chlorides, nitrates, nitrites, sulphates and aluminum.

The pH, TS, VS and COD were measured following standard methods (APHA 2005). COD was determined following Method 5220 D (closed reflux, colorimetric method) using a Perkin Elmer Lambda 35 Visible – UV system. The determination of F^-^, Cl^-^, NO_3_^-^, NO_2_^-^ and SO_4_^2-^ was carried out by ionic chromatography (861 Advanced Compact IC 2.861.0010), after filtering the wastewater with a 0.45-μm pore size filter. Al was determined by inductively coupled plasma mass spectrometry (ICP-MS), after acid digestion.

### Analysis of experimental data

#### Equilibrium study

The equilibrium sorption capacities of the resins were determined using the following expression:

1 $${\mathrm{q}}_{\mathrm{eq}}=\left({\mathrm{C}}_0-{\mathrm{C}}_{\mathrm{eq}}\right)\cdot \frac{\mathrm{V}\ }{\mathrm{M}}$$

where: q_eq_ is the equilibrium sorption capacity of the resin for fluoride (mg F^-^/g resin); V is the volume of solution (L); M is the resin weight (g); C_0_ and C_eq_ are the initial and equilibrium concentrations of fluoride (mg F^-^/L), respectively. C_eq_ is estimated in the kinetic study (see “Generalized integrated Langmuir kinetic equation” section).

#### Kinetic study

##### Generalized integrated Langmuir kinetic equation

To obtain the values of the equilibrium constants and concentrations, the generalized integrated Langmuir kinetic equation (Marczewski et al. 2013) was used:

2 $$\mathrm{C}={\mathrm{C}}_0+\mathrm{F}\cdot \left({\mathrm{C}}_{\mathrm{eq}}-{\mathrm{C}}_0\right)$$

where: C is the concentration (mg F^-^/L) and F is the sorption progress variable.

The generalized integrated Langmuir kinetic equation was obtained using the experimental data and Microsoft Excel Solver. Then, other model parameters were obtained using the following expressions:

3 $$\mathrm{F}=\frac{1-\exp \left(-{\mathrm{k}}_{\mathrm{L}}\mathrm{t}\right)}{1-{\mathrm{f}}_{\mathrm{L}}\exp \left(-{\mathrm{k}}_{\mathrm{L}}\mathrm{t}\right)}$$

4 $${\mathrm{k}}_{\mathrm{L}}=\frac{\mathrm{A}}{1-{\mathrm{f}}_{\mathrm{L}}}$$

5 $$\mathrm{A}={\left(\frac{\mathrm{dF}}{\mathrm{dt}}\right)}_{\mathrm{F}=0}$$

k_L_ is the rate coefficient (always positive), f_L_ is the generalized Langmuir batch equilibrium factor, which is positive for sorption conditions (0 ≤ f_L_ < 1), and A is the initial sorption relative progress rate (A > 0).

Another parameter of interest when comparing resins is the sorption halftime time, τ, which is the time at which F = 0.5, i.e. the time necessary to reach half of C_0_ (50% of the maximum capacity). It is expressed as follows (Marczewski et al. 2013):

6 $$\uptau =\frac{\mathrm{t}}{{\mathrm{t}}_{1/2}}$$

If f_L_ < 1, t_1/2_ is calculated with the expression:

7 $${\mathrm{t}}_{1/2}=\ln \frac{2-{\mathrm{f}}_{\mathrm{L}}}{{\mathrm{k}}_{\mathrm{L}}}$$

Three graphical representations of the experimental results are of interest in this study:

- Fluoride concentration in the treated ECWT versus time, to assess the effect of dosage using the resins doped with aluminum (i.e. Al-TP260 and Al-TP207), the trend being modeled with the generalized integrated Langmuir kinetics using the ‘solver’ function of Microsoft Excel.
- Ion exchange progress (F) versus τ. For the exchange process to be favorable, it should be above a hypothetical bisector that represents f_L_ = 0 (Marczewski et al. 2013). The slope of the hypothetical bisector varies depending on the kinetic model obtained for a specific resin and dosage.
- Ion exchange progress (F) versus compact time (τ/(1+ τ)). The best option would be that the rate of exchange of fluorides were constant, i.e. a straight line. Thus, less favorable processes are those whose curves above or below the bisector (Marczewski et al. 2013).

##### Pseudo-first-order and pseudo-second-order kinetic models

Pseudo-first-order and second-order kinetic models were used to describe the experimental data, using Eq. (8) and Eq. (9), respectively. Both expressions have been widely used: the first one was created by Langergren and the second one is one the most applied linear pseudo-second-order kinetic models (Meenakshi and Viswanatha 2007; Shin et al. 2021):

8 $$\log \left(\frac{{\mathrm{q}}_{\mathrm{eq}}}{{\mathrm{q}}_{\mathrm{eq}-}{\mathrm{q}}_{\mathrm{t}}}\right)=\frac{\mathrm{k}^{\prime }}{2.303}.\mathrm{t}$$

9 $${\mathrm{q}}_{\mathrm{t}}=\frac{{\mathrm{q}}_{\mathrm{eq}}^2.\mathrm{k}.\mathrm{t}}{1+{\mathrm{q}}_{\mathrm{eq}}.\mathrm{k}.\mathrm{t}}$$

where: q_t_ and q_eq_ are the loading capacities at time t and equilibrium (mg F^-^/g resin), respectively; k’(min^-1^) and k (g F^-^·mg^-1^·min^-1^) are the pseudo- first-order and second-order sorption rate constants, respectively.

#### Isotherm study

##### Langmuir isotherm model

The Langmuir equilibrium isotherm can be expressed as (Kameda et al. 2015):

10 $${\mathrm{q}}_{\mathrm{eq}}=\frac{{\mathrm{K}}_{\mathrm{L}}\cdot {\mathrm{q}}_{\mathrm{max}}\cdot {\mathrm{C}}_{\mathrm{eq}}}{1+{\mathrm{K}}_{\mathrm{L}}\cdot {\mathrm{C}}_{\mathrm{eq}}}$$

where, q_max_ is the maximum sorption capacity (mg F^-^/g resin) and K_L_ is the Langmuir constant related to sorption energy.

##### Freundlich isotherm model

The Freundlich equilibrium isotherm can be expressed as (Li et al. 2020):

11 $${\mathrm{q}}_{\mathrm{eq}}={\mathrm{K}}_{\mathrm{F}}\cdot {\mathrm{C}}_{\mathrm{eq}}^{1/\mathrm{n}}$$

where, K_F_ is the Freundlich sorption capacity constant, and 1/n is a characteristic constant related to sorption intensity (Bhatt et al. 2004).

## Results and discussion

### Physicochemical characterization of the coke wastewater

Table 2 shows the results of the physicochemical characterization of the ECWT before and after the filtration. Solids were found to be mainly inorganic, given that < 3% of TS are VS, which are linked to organic matter. The value of COD, representing the organic matter present in the wastewater, was similar before and after the filtration. The reduction of fluoride before and after filtration can be related to the complex matrix of ammonia water and the presence of fluoride-related forms to in the particulate matter present in the water. Similarly, the concentration of other co-existent anions showed a variation due to this process, the higher differences being observed in the case of Cl^-^ and SO_4_^2-^.

**Table 2:** Results of the initial physicochemical characterisation of the ECWT (*n* = 3)

Parameter	Before filtration	After filtration
pH	7.20 ± 0.06	7.18 ± 0.04
COD (mg/L)	346.75 ± 26.31	323.17 ± 23.47
TS (g/L)	12.22 ± 0.53	BDL
VS (g/L)	0.35 ± 0.06	BDL
Chlorides (mg Cl^-^/L)	2613.0 ± 160.5	1903.8± 134.0
Fluorides (mg F^-^/L)	31.00 ± 0.51	26.70 ± 0.15
Nitrates (mg NO_3_^-^/L)	131.4 ± 55.7	132.88 ± 13.96
Nitrites (mg NO_2_^-^/L)	2.00 ± 1.50	1.44 ± 0.01
Sulphates (mg SO_4_^2-^/L)	7950.0 ± 411.5	6058.1 ± 213.6

*BDL* below detection limit

### Experimental results: effect of dosage

**Fig. 1:**
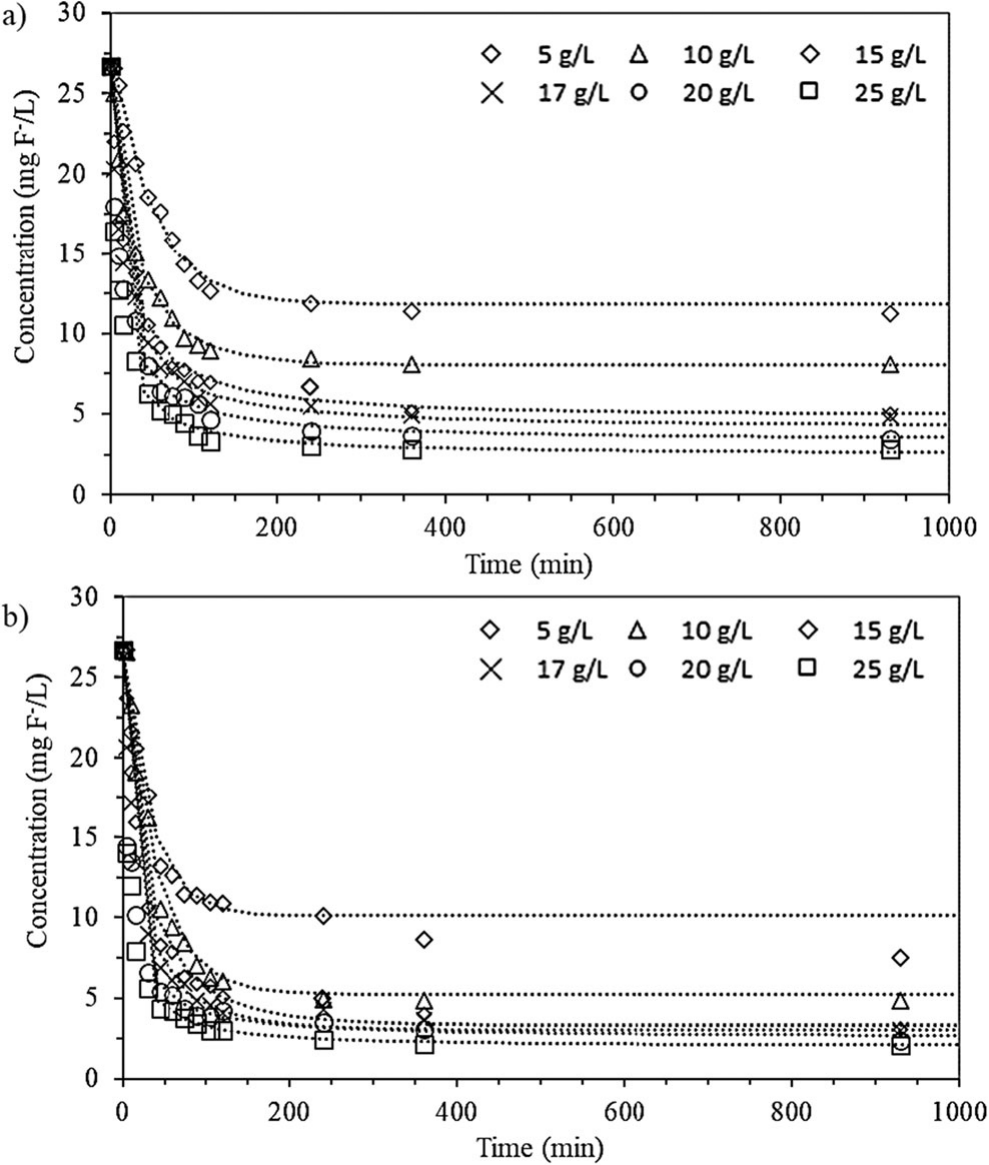
Fluoride concentration, in mg F^-^/L versus time, using different dosages (g of resin per L of wastewater) of Al-doped TP260 (**a**) and TP207 (**b**) resins. Trends (dotted lines) are calculated using the generalized integrated Langmuir kinetics

Figure 1 shows the results obtained throughout the ion exchange processes carried out with different dosages of Al-doped TP260 and TP207 resins. Fluoride concentrations decreased over time at higher rates of sorption at the beginning of the ion exchange process. After around 2 h, equilibrium was reached regardless of the resin. By that time, with the highest resin dosage (25 g/L), the concentration of fluoride was reduced by more than 88% (from 26.7 mg F^-^/L to < 3 mg F^-^/L) with both resins.

**Fig. 2:**
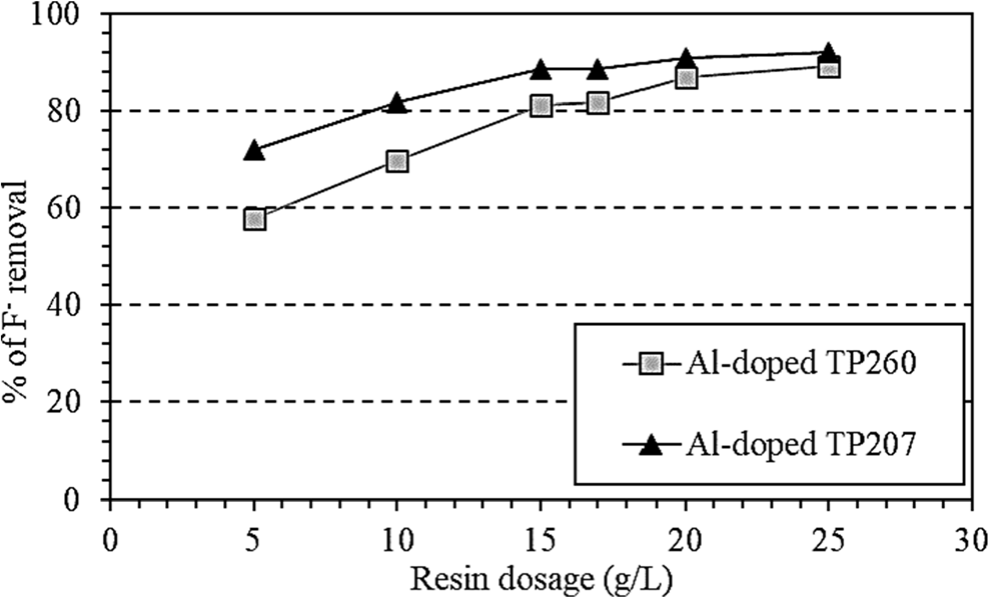
Percentage of F^-^ removal after the ion exchange process using different dosages (g of resin per L of wastewater) of Al-doped TP260 and TP207 resins

Figure 2 shows the percentage of fluorine removal achieved with the two Al-doped resins. F^-^ removal was within the range 57.8–89.3% and 72.0–92.1% for Al-doped TP260 and TP207, respectively. The lowest final F^-^ concentration (2.1 mg F^-^/L) was achieved with 25 g/L of Al-doped TP207 resin (92.1% of F^-^ removal). The differences observed between the two tested resins when the same dosage was applied may be related to the type of functional group present in the material, as observed by Li et al. (2020).

Increasing the dosage of Al-doped TP207 resin from 15 to 20 g/L and from 20 to 25 g/L led to slight improvements in the F^-^ removal (2.9% and 1.2%, respectively). However, the results obtained with the Al-doped TP260 resin showed a 7.1% increase in F^-^ removal with a dosage of 20 g/L compared to 15 g/L and 2.7% higher when using a resin dosage of 25 g/L instead of 20 g/L. Thus, for the higher resin dosages tested, it seems that the ion exchange process was influenced more by the ratio resin:wastewater when using Al-doped TP260 resin than using Al-doped TP207 resin.

Meenakshi and Viswanatha (2007) tested a chelating resin with sulfonic acid functionality and an anion exchange resin in Cl^-^ form in batch conditions and found that the F^-^ removal efficiency increased with the dosage of the resin (tested dosage 10–60 g/L). The initial concentration of fluoride in the wastewater used in their experiments was 3 mg F^-^/L and the highest F^-^ removal was around 75% with the chelating resin at a dosage of 60 g/L. Nonetheless, with 10 g/L of each resin, the treated effluents had concentrations below 1 mg F^-^/L. The differences observed in the required resin dosage with respect to the present study may be related not only to the type of tested resin in both studies, but also to the initial concentration of F^-^ in the wastewater, given that the sorption process can be affected by several parameters, such as sorbent dosage, pH, contact time, initial fluoride concentration, dissolved salts, and temperature (Bhatt et al. 2004).

It is important to note that the process efficiency may be affected by coexisting anions (e.g. Cl^-^, NO_3_^-^) that may compete with F^-^ depending on their concentration and affinity for the exchanger (Tao et al. 2020). In the ECWT used in this study, some anions were present in high concentrations (Table 2), which may reduce the defluorination capacity of the resins if they are exchanged first than F^-^. Future research should assess this effect on the process at different concentrations of co-ions, as well as organic matter, in the wastewater. Dixit et al. (2020) observed 70% removal of organic matter in an ion-exchange process and pore blockage of the resins depending on the molecular weight of this fraction in water. Gönder et al. (2006) obtained capacity losses up to 30% in ion-exchange resins exposed to organic materials.

Attention must be paid when using metal-based exchangers such as those used in the present work, given that metal leakages may occur during the process. It depends on the success of the doping process, but also the chemical species considered. For instance, Shin et al. (2021) found that AMPA chelating resins doped with Al presented more leakage than those doped with Zr when used in column trials for fluoride removal.

Table 3 shows the Al concentration in the ECWT after the ion exchange process for the tests performed with the three highest resin dosages. Al concentration was found to be 59.2 ± 4.2% higher when using Al-doped TP207 resin instead of Al-doped TP260 resin, up to 64% more leaching for the same resin dosage. These results may suggest a weaker attachment of Al^3+^ to iminodiacetic groups than to AMPA groups under the same doping process. In all the studied cases, the aluminum content in the ion exchange effluents was found to be < 0.4 mg Al/L, below the minimum acceptable limits.

**Table 3:** Aluminum concentration in the ECWT after the ion exchange process using Al-doped TP260 and TP207 resins

Dosage (g resin/L ECWT)	Al-doped TP260 resin	Al-doped TP207 resin
	Concentration (μg Al/L)	
17	240.3	380.0
20	225.9	370.2
25	231.8	360.6

### Kinetic study results

#### Results of the generalized integrated Langmuir kinetic model

Figure 1 shows the trends that fit the experimental data obtained with the generalized integrated Langmuir kinetic equation, as explained in “Generalized integrated Langmuir kinetic equation” section. Table 4 shows the values obtained for the parameters of the model for the six dosages of the two Al-doped ion exchange resins that were tested. The R-squared coefficients revealed that good agreement between the experimental data and the kinetic model was achieved (R^2^ > 0.98). Furthermore, as expected in a sorption process, k_L_ values were always positive, and the generalized Langmuir batch equilibrium factor was 0 ≤ f_L_ < 1.

**Table 4:** Calculated values of the parameters of the generalized integrated Langmuir kinetic equation for Al-doped TP260 and TP207 resins and the R-squared coefficients

Parameters	Dosage of Al-doped TP260 (g resin/L)					
	5	10	15	17	20	25
C_eq_ (mg F^-^/L)	11.24	7.05	4.95	4.77	3.35	2.54
k_L_	0.017	0.007	0.003	0.006	0.0006	0.001
f_L_	0.000	0.823	0.953	0.914	0.994	0.991
R^2^	0.991	0.986	0.991	0.995	0.993	0.997
t_1/2_ (min)	39.55	22.14	15.63	12.84	10.63	7.56
Parameters	Dosage of Al-doped TP207 (g resin/L)					
	5	10	15	17	20	25
C_eq_ (mg F^-^/L)	10.28	5.21	4.12	3.69	2.64	2.00
k_L_	0.033	0.026	0.021	0.022	0.0003	0.001
f_L_	0.000	0.000	0.584	0.686	0.998	0.994
R^2^	0.983	0.985	0.993	0.997	0.990	0.995
t_1/2_ (min)	20.92	26.83	16.24	12.23	6.44	5.43

**Fig. 3:**
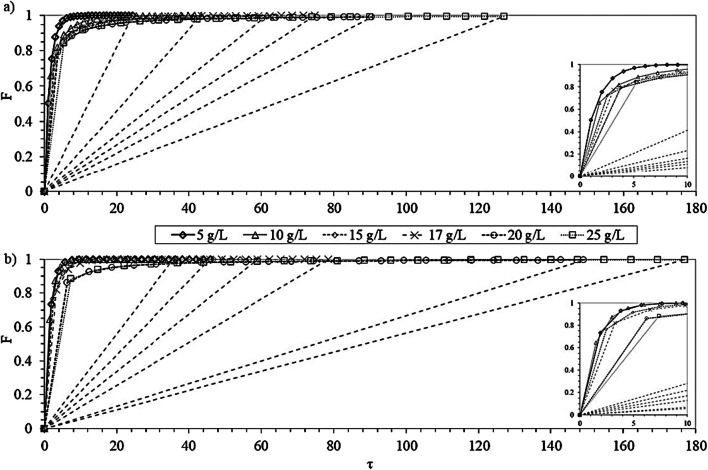
Ion exchange progress (F) versus the sorption halftime time (τ) using different dosages (g resin/L) of Al-doped TP260 (**a**) and TP207 (**b**) resins. Bisectors are plotted as dashed lines without markers

Figure 3 represents F versus τ for the two Al-doped resins. All the curves obtained have a positive tendency and are on the left side of the hypothetical bisector (f_L_ = 0), which means that for any equilibrium concentration, the process was favorable for both resins. When designing a column test, it would be necessary to work above τ to guarantee a good solid-liquid contact.

**Fig. 4:**
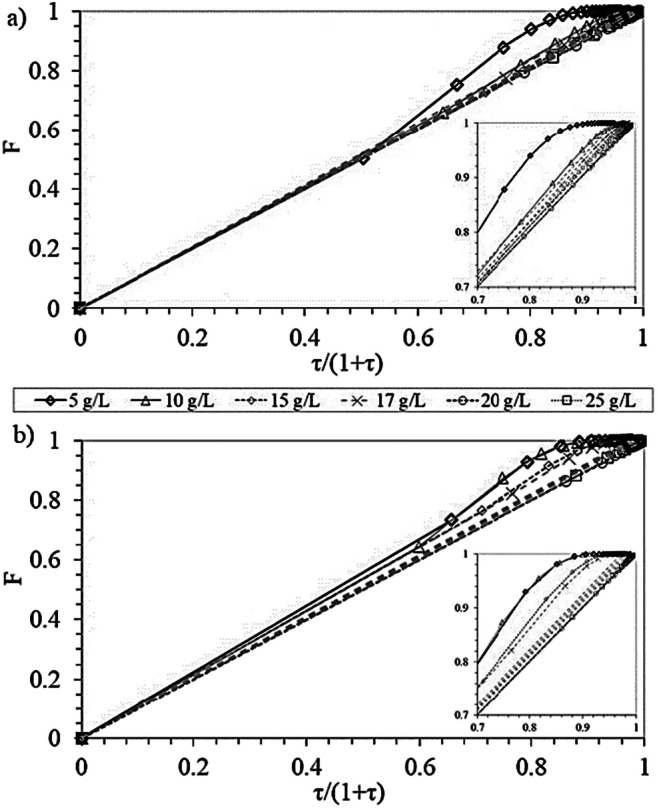
Ion exchange progress (F) versus compact time (τ/(1+τ)) using different dosage (g resin/L) of Al-doped TP260 (**a**) and TP207 (**b**) resins. Bisectors are plotted as dashed lines without markers

Figure 4 represents F versus the compact time calculated for both resins. For the same value of τ and low τ/(1+τ) values (< t_1/2_) and regardless of the resin dosage, the sorption progress with Al-doped TP260 was close to a straight line, which means that the F^-^ exchange rate was constant. By contrast, in the case of Al-doped TP207 resin, small differences between the resin dosages can already be observed. For the highest compact times, differences became more evident with both resins. In general, Al-doped TP260 resin was more favorable than Al-doped TP207 resin, given that most of its curves are closer to a straight line, except for the lowest dosages (< 10 g/L). In the case of Al-doped TP207, resin dosages of 20 and 25 g/L seemed to have a similar sorption progress at a constant F^-^ exchange rate.

#### Results of pseudo-first-order and pseudo-second-order kinetic models

The graphical representations of q_t_ versus time obtained using the pseudo-first-order and pseudo-second-order kinetic models are included in Figures S3 and S4 (Online Resource 1). Figure S5 (Online Resource 1) represents t/qt versus time, which is used to calculate k and q_eq_ from the slopes and intercepts. Table 5 shows the parameters of the models that were obtained for each resin and dosage tested. The two kinetic models fit well the experimental data obtained with the two Al-doped resins, given the high R-squared obtained.

**Table 5:** Calculated values of the parameters and the R-squared coefficients of the pseudo-first-order and pseudo-second-order kinetic models for Al-doped TP260 and TP207 resins

Pseudo-first-order kinetic model						
Parameters	Dosage of Al-doped TP260 (g/L)					
	5	10	15	17	20	25
q_eq_ (mg F^-^/g resin)	3.15	1.81	1.34	1.22	1.09	0.91
k’ (min^-1^)	0.016	0.034	0.046	0.055	0.070	0.092
R^2^	0.995	0.993	0.989	0.990	0.986	0.990
Parameters	Dosage of Al-doped TP207 (g/L)					
	5	10	15	17	20	25
q_eq_ (mg F^-^/g resin)	3.59	2.23	1.45	1.30	1.14	0.94
k’ (min^-1^)	0.026	0.024	0.042	0.056	0.103	0.116
R^2^	0.992	0.994	0.997	0.998	0.990	0.993
Pseudo-second-order kinetic model						
Parameters	Dosage of Al-doped TP260 (g/L)					
	5	10	15	17	20	25
q_e_ (mg F^-^/g resin)	3.29	1.96	1.49	1.33	1.18	0.98
k (g·mg^-1^·min^-1^)	0.010	0.030	0.042	0.057	0.083	0.136
R^2^	0.974	0.989	0.996	0.997	0.996	0.998
Parameters	Dosage of Al-doped TP207 (g/L)					
	5	10	15	17	20	25
q_e_ (mg F^-^/g resin)	3.92	2.30	1.60	1.45	1.21	1.00
k (g·mg^-1^·min^-1^)	0.010	0.030	0.038	0.053	0.130	0.185
R^2^	0.986	0.997	0.992	0.997	0.995	0.998

### Results of Langmuir and Freundlich isotherm models

Table 6 contains the values of the calculated parameters of the Langmuir and Freundlich isotherms for both tested Al-doped resins. The highest values of q_max_, i.e. the highest maximum sorption capacities, were achieved with the Al-doped TP260 resin, reaching 32.06 mg F^-^/g resin (C_0_ = 26.70 mg F^-^/L; pH = 7.18). According to Bhatnagar et al. (2011), other authors have reported similar values using zeolite-Al^3+^ and zeolite-La^3+^ (C_0_ = 10–80 mg F^-^/L, sorption capacity 28–41 mg F^-^/L), alum-impregnated activated alumina (C_0_ = 1–35 mg F^-^/L, sorption capacity 40.68 mg F^-^/L) and hydrated iron(III)–aluminium(III)–chromium(III) ternary mixed oxide (C_0_ = 10–80 mg F^-^/L, sorption capacity 31.89 mg F^-^/L). Millar et al. (2017) described experimental data for the removal of fluoride ions by chelating ion exchange resins with iminodiacetate functionality pre-treated with a solution of aluminum chloride prior to use. According to the Langmuir model, their estimated maximum loadings were 1.3, 12.4, and 60.7 mg F^-^/g resin for initial fluoride concentrations of 10, 100 and 1000 mg F^-^/L, respectively, at a solution pH of 6–7.5.

**Table 6:** Calculated values of the parameters of Langmuir and Freundlich isotherm models from experimental data of ion exchange processes using Al-doped TP260 and TP207 resins

	Parameters	Al-doped TP260	Al-doped TP207
Langmuir	q_max_ (mg F^-^/g resin)	32.06	12.05
	K_L_	0.010	0.039
	R^2^	0.985	0.990
Freundlich	K_F_	0.345	0.525
	1/n	0.914	0.808
	R^2^	0.987	0.991

As seen, Langmuir isotherm is commonly applied to describe adsorption and ion-exchange processes. Both ion exchange and adsorption are surface phenomenon in which dissolved chemical species are taken up by a solid. However, in ion exchange processes, free mobile ions on a solid water-insoluble substance (i.e. cation or anion exchanger, depending on the ionic groups attached) are stoichiometrically replaced by different ions of similar charge present in the aqueous medium with which it is in contact, whereas in adsorption processes, the chemical species are captured without any exchange (Kumar and Jain 2013).

Berber-Mendoza et al. (2006) compared the ion exchange isotherm, which is based on the constant of thermodynamic equilibrium for the ion exchange reaction, and the Langmuir isotherm. The experimental data obtained in an ion exchange process using natural zeolites to remove Pb(II) fitted both isotherms but the latter was found to be simpler in use, as only two constants have to be estimated.

**Fig. 5:**
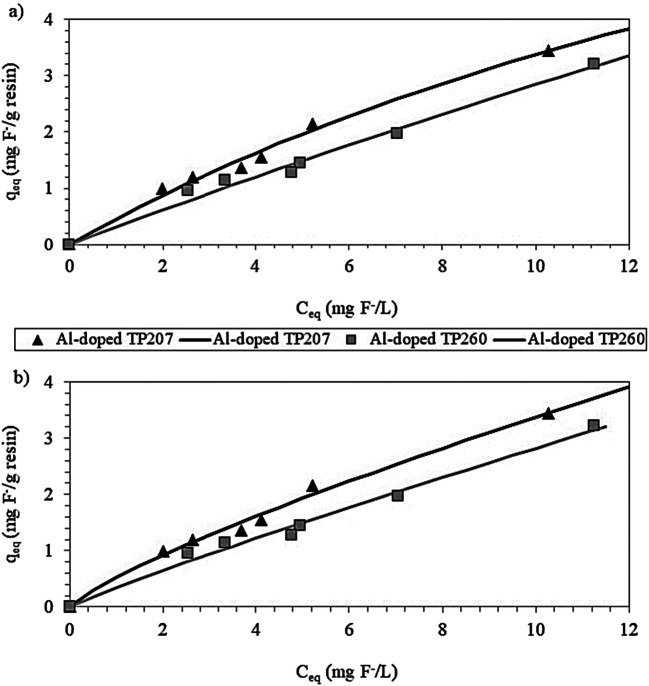
Calculated Langmuir (**a**) and Freundlich (**b**) isotherms (lines) for experimental data (markers) obtained for ion exchange processes using Al-doped TP260 and TP207 resins

Figure 5 shows the graphical representation of q_eq_ versus C_eq_, which were obtained in the kinetic study for each resin dosage (see Table 4). The calculated isotherms follow an upward curve with good fit to experimental data (R^2^ > 0.98), regardless of the resin. The R-squared obtained with the Langmuir model was slightly higher than with the Freundlich model for Al-doped TP260, whereas the opposite was observed for Al-doped TP207 resin (Table 6). Nonetheless, these differences in the regression values are not enough to provide correct conclusions about the surface heterogeneity of each resin tested (Kónya and Nagy 2013). According to these authors, isotherms not being linear (Figure 5) means that there is a competing ion, which is something that happens in a competitive adsorption process and in an ion exchange process. Thus, the isotherms could be divided in different linear portions, meaning that a heterogenous surface could be treated as composed by homogeneous portions.

Furthermore, the values of 1/n of the Freundlich isotherm model were between 0 and 1 for both Al-doped resins (Table 6), which indicates favorable sorption (Rengaraj et al. 2007). Besides, when 1/n < 1, the sorption surface may be heterogenous and/or the sorbed species may be rejected and when 1/n > 1, the surface is homogeneous and the sorbed species attract each other (Kónya and Nagy 2013). In the present study, Al-doped TP260 presented a greater 1/n value and closer to 1 than Al-doped TP207 (see Table 6).

## Conclusions

Ion exchange technology was studied with the objective of reducing the fluoride concentration in the effluent of a coke wastewater treatment plant (C_0_ = 26.70 ± 0.15 mg F^-^/L). Two Al-doped exchange resins with chelating AMPA and iminodiacetic groups (Al-doped TP260 and TP207 resins, respectively) were assessed. The effect of resin dosage was evaluated, varying from 5 to 25 g/L. F^-^ removal was within the range 57.8–89.3% and 72.0–92.1% for Al-doped TP260 and TP207, respectively. The highest dosage of the latter reported the lowest final F^-^ concentration (2.1 mg F^-^/L). In general, a higher influence of resin dosage was observed with the Al-doped TP260 resin.

The generalized integrated Langmuir kinetic equation fitted the experimental data (R^2^ > 0.98), allowing it to be verified that the parameters of said kinetics meet the optimal conditions for the process. In general, using the same dosage, the ion exchange process seemed to be more favorable with Al-doped TP260 resin than with Al-doped TP207 resin, given that the F^-^ exchange rate was more constant. Furthermore, the experimental data were well described (R^2^ > 0.98) by Langmuir and Freundlich isotherm models. The maximum sorption capacity was obtained for Al-doped TP260 resin, in agreement with the findings of the kinetic study.

## Supplementary Information

ESM 1 (DOCX 290 kb)

## Data Availability

All data generated or analysed during this study are included in this published article.
