# Supplementary material for: Removal of fluoride from coke wastewater by aluminum doped chelating ion-exchange resins: a tertiary treatment
Source: Environ Sci Pollut Res Int. 2021 Sep 7;29(6):8705–15. doi: 10.1007/s11356-021-16299-8 (PMC8776662; doi:10.1007/s11356-021-16299-8)
Supplement: Supplementary file 1 — (DOCX 290 kb) [file 11356_2021_16299_MOESM1_ESM.docx]

**Supplementary material**

**REMOVAL OF FLUORIDE FROM COKE WASTEWATER BY ALUMINUM DOPED CHELATING ION-EXCHANGE RESINS: A TERTIARY TREATMENT**

Jesús Rodríguez-Iglesias ^1^, Lara Alcalá ^1^, Laura Megido ^1*^, Leonor Castrillón ^1^

^1^Department of Chemical and Environmental Engineering, Polytechnic School of Engineering, Gijón Campus, University of Oviedo, 33203 Gijón, Spain

*Corresponding author: megidolaura@uniovi.es; Tel.: (+34) 985 182 026; Fax: (+34) 985 182 337

**Environmental Science and Pollution Research**

**S.1. Introduction**

Table S1 reviews some results found in the literature of the application of ion exchange resins to reduce the concentration of fluorides in synthetic and real water samples.

Table S1. Defluorination capacity of ion exchange resins found in literature.

| **Ion-exchange resin** | **Concentration (mg F^-^/L)** | | **Defluorination capacity** | **pH** | **Reference** |
| --- | --- | --- | --- | --- | --- |
|  | **Initial** | **Final** |  |  |  |
| Fe^3+^-type chelating resins containing different functional groups | 15  (synthetic sample) | >5 | < 1 g F^-^/kg resin | 5.2 | Li et al. (2020) |
| Chelating resin with sulfonic acid functionality (Indion FR 10^a^) | 2.79  (field sample) | 0.83 | 0.098 g F^-^/kg resin | 7.7 | Meenakshi and Viswanathan (2007) |
|  | 3  (synthetic sample) | 1 | 0.100 g F^-^/kg resin | 3 - 11 |  |
| Anion exchange resin in Cl^-^ form (Ceralite IRA 400) | 2.79  (field sample) | 1.83 | 0.048 g F^-^/kg resin | 8.4 |  |
|  | 3  (synthetic sample) | 1 | < 0.07 g F^-^/kg resin | 3 - 11 |  |
| Al-doped chelating resins | 9  (field sample) | < 1 | 2.6 g F^-^/L resin | 5.8 | Oke et al. (2011) |
| Al pre-loaded chelating resin with AMPA group | 25  (synthetic sample) |  | < 6.5 g F^-^/kg resin | 7 | Shin et al. (2021) |
| Zr pre-loaded chelating resin with AMPA group | 25  (synthetic sample) |  | < 3.5 g F^-^/kg resin |  |  |

**S.2. Materials and methods**

Figure S1 shows how the AMPA and iminodiacetic acid groups of the ion-exchange resins are doped with Al using a solution of AlCl_3_. Figure S2 shows the replacement of Cl^-^ in the resin by the F^-^ removed from the wastewater during the ion exchange process.

Functional group: iminodiacetic acid


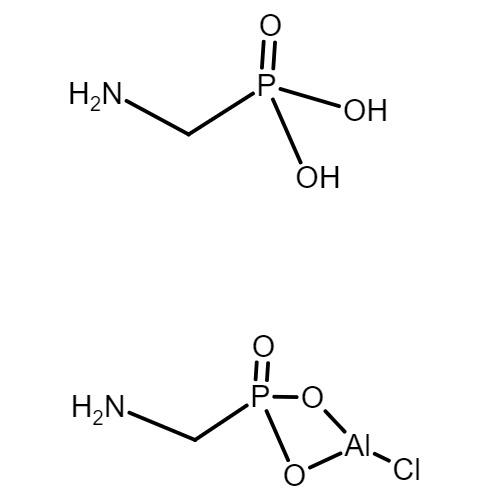

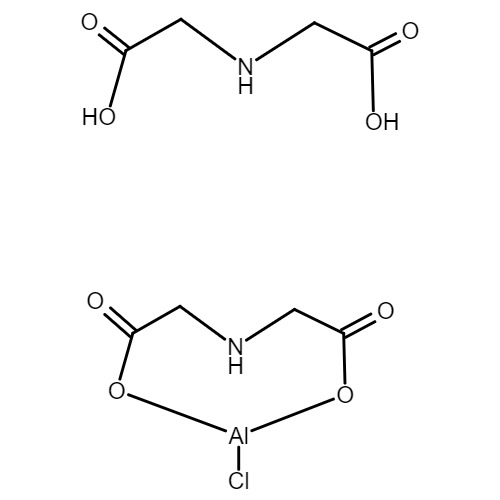

$$\boldsymbol{+}\mathbf{AlCl}_{\mathbf{3}}$$

Functional group: AMPA

$$\boldsymbol{+}\mathbf{AlCl}_{\mathbf{3}}$$

Figure S1. AMPA and iminodiacetic acid groups doped with Al using a solution of AlCl_3_.

Functional group: iminodiacetic acid

Functional group AMPA


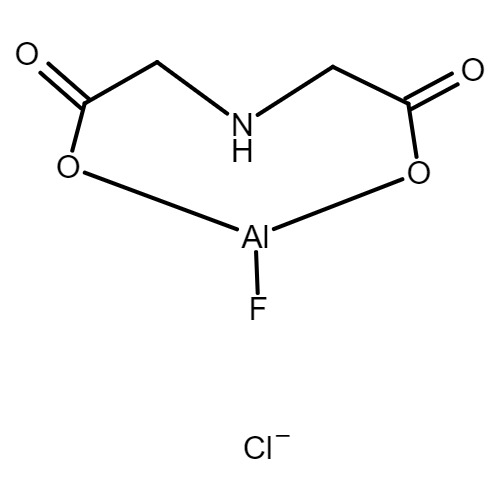

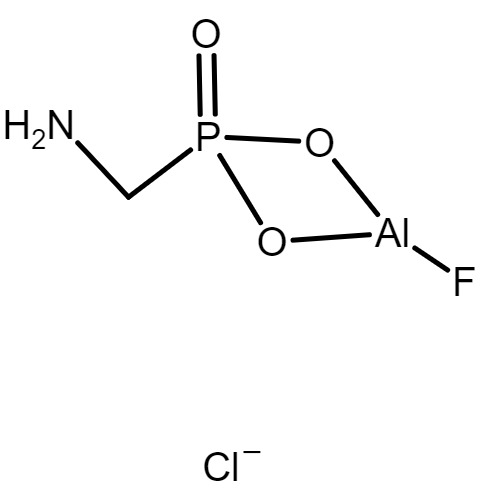

$$+$$

$$+$$

Figure S2. Replacement of Cl^-^ by F^-^ in Al-doped AMPA and iminodiacetic acid groups.

**S.3. Results**

*S.3.1. Results of the pseudo-first-order and pseudo-second-order kinetic model*

Figures S3 and S4 show the modelisation of the experimental data obtained with the two Al-doped resins under study, using a pseudo-first-order and a pseudo-second-order kinetic models, respectively. Figure S5 represents t/q_t_ versus time, which is used to calculate k, q_eq_ and the initial sorption rate h=k·q_eq_^2^ from the slopes and intercepts (Meenakshi and Viswanathan 2007).

a)

b)

Figure S3. Graphical representation of q_t_ (mg F^-^/g resin) versus time (min) obtained using different dosages of Al-doped TP207 (a) and Al-doped TP260 (b). Continuous lines represent the pseudo-first-order kinetic model that fits the experimental data.

a)

b)

Figure S4. Graphical representation of q_t_ (mg F^-^/g resin) versus time (min) obtained using different dosages of Al-doped TP207 (a) and Al-doped TP260 (b). Continuous lines represent the pseudo-second-order kinetic model that fits the experimental data.

a)

b)

Figure S5. Graphical representation of t/q_t_ (min·g resin/mg F^-^) versus time (min) obtained using different dosages of Al-doped TP207 (a) and Al-doped TP260 (b). Continuous lines represent the pseudo-second-order kinetic model that fits the experimental data.

**References**

Li R, Tian X, Ashraf I, Chen B (2020) Fluoride removal using a chelating resin containing phosphonic-sulfonic acid bifunctional group. J Chromatogr A 1613:460697. https://doi.org/10.1016/j.chroma.2019.460697

Meenakshi S, Viswanatha N (2007) Identification of selective ion exchange resin for fluoride sorption. J Colloid Interface Sci 308:438–450. https://doi.org/10.1016/j.jcis.2006.12.032

Oke K, Neumann S, Adams B (2011) Selective elimination of fluorine. Water Today. 76-80.

Shin E, Dreisinger DB, Burns AD (2021) Removal of fluoride from sodium sulfate brine by zirconium pre-loaded chelating resins with amino-methyl phosphonic acid functionality. Desalination 505:114985. https://doi.org/10.1016/j.desal.2021.114985
